# Supplementary material for: Synthesis of Carvone Derivatives and In Silico and In Vitro Screening of Anti-Inflammatory Activity in Murine Macrophages
Source: Int J Mol Sci. 2023 Jan 23;24(3):2263. doi: 10.3390/ijms24032263 (PMC9916994; doi:10.3390/ijms24032263)
Supplement: Supplementary file 1 [file ijms-24-02263-s001.zip › ijms-2168159-supplementary.pdf]

Supplementary Materials to "Synthesis of Carvone Derivatives and *In Silico* and *In Vitro* Screening of Anti-Inflammatory Activity in Murine Macrophages" by Gabriela Moço, Cátia Sousa, Ana Capitão, Stephen Scott MacKinnon, Alcino Leitão and Alexandrina Ferreira Mendes

NO Production

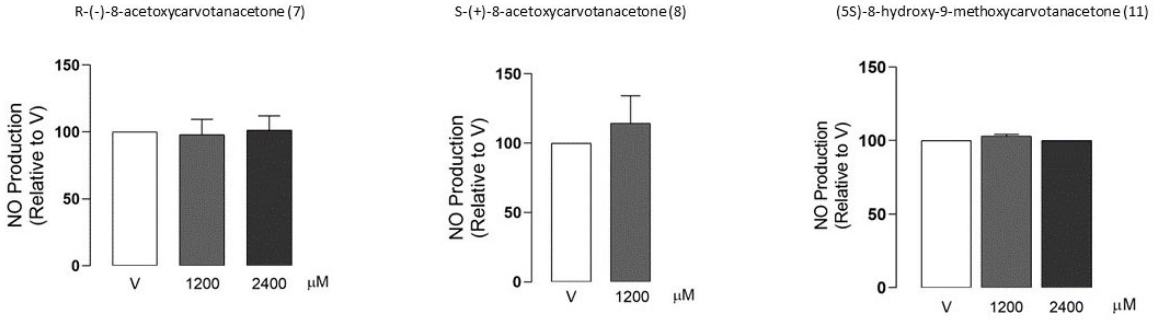

Figure S1. Effect of the test compounds on NO production. Raw 264.7 cells were treated with 1200 or 2400 μM of the test compounds for 18 h in the absence of LPS; control cells (V) were treated with the vehicle alone (0,1% DMSO) for 18 h. Each column represents the mean ± SEM of three independent experiments.

NMR (^{1}H and ^{13}C) and FT-IR Spectroscopy

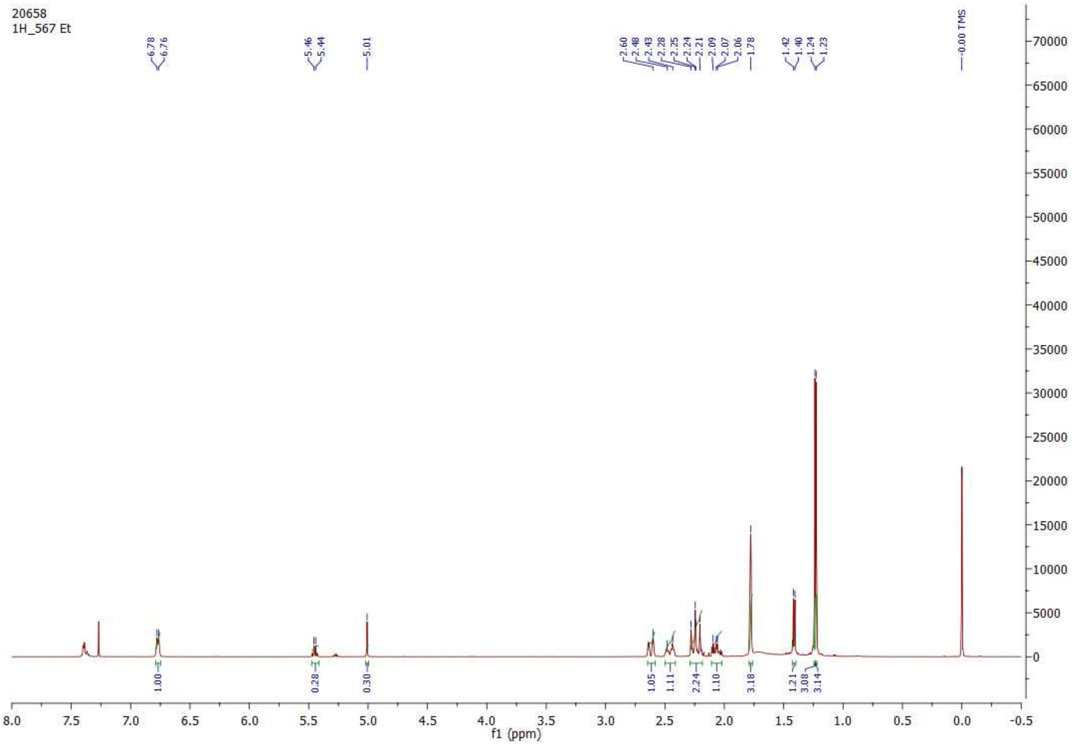

Figure S2. ^{1}H NMR spectrum of **3**.

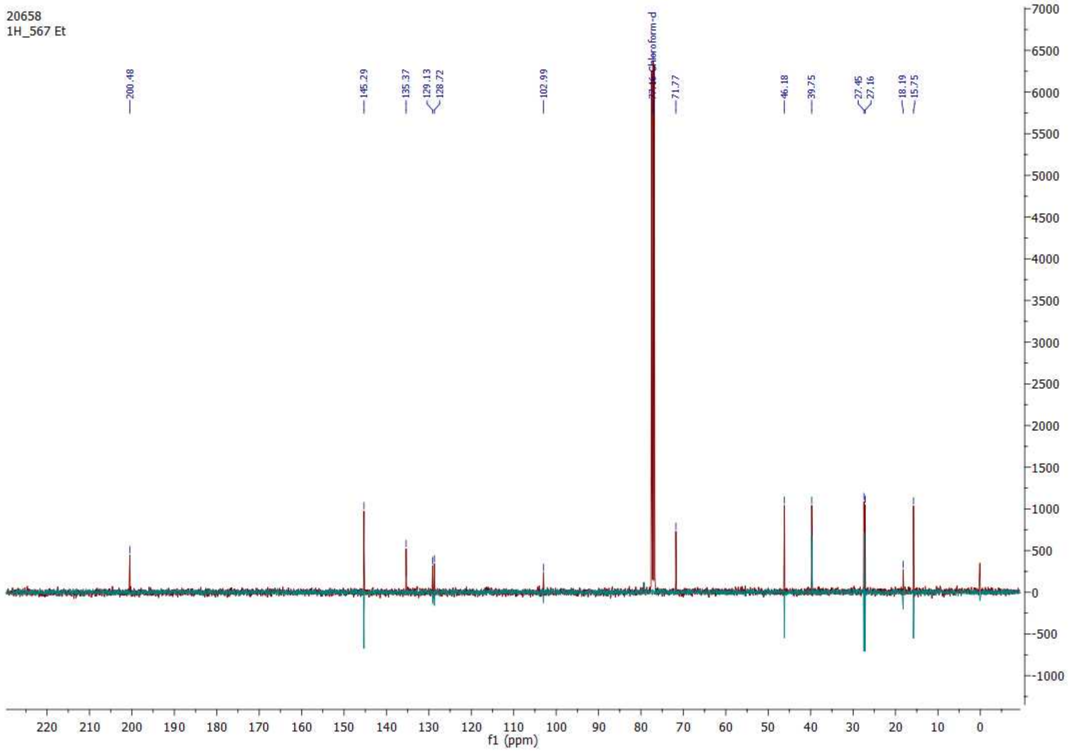

Figure S3. ^{13}C NMR and DEPT-135 spectra of **3.**

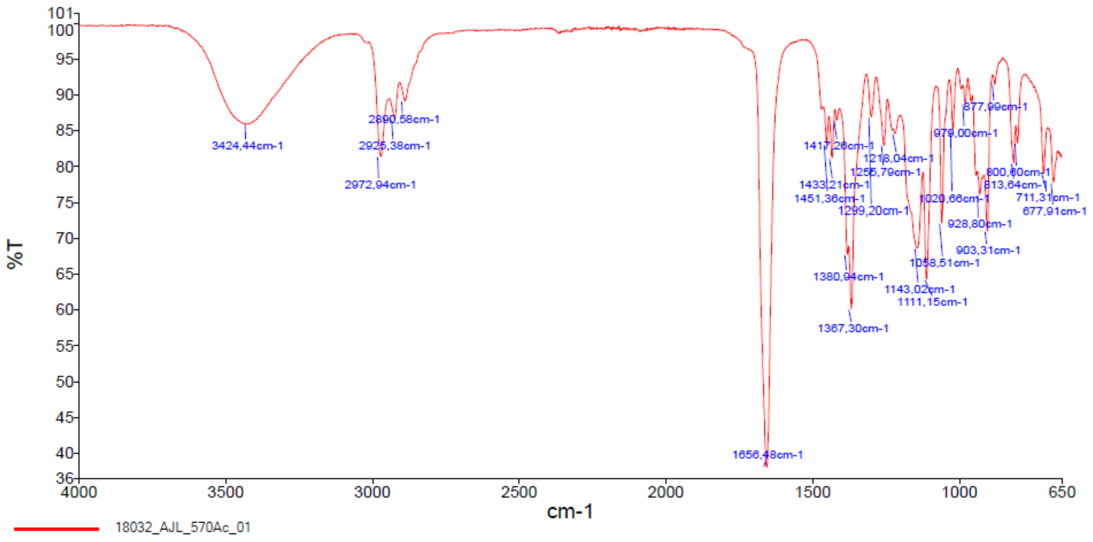

Figure S4. FT-IR spectrum of **3.**

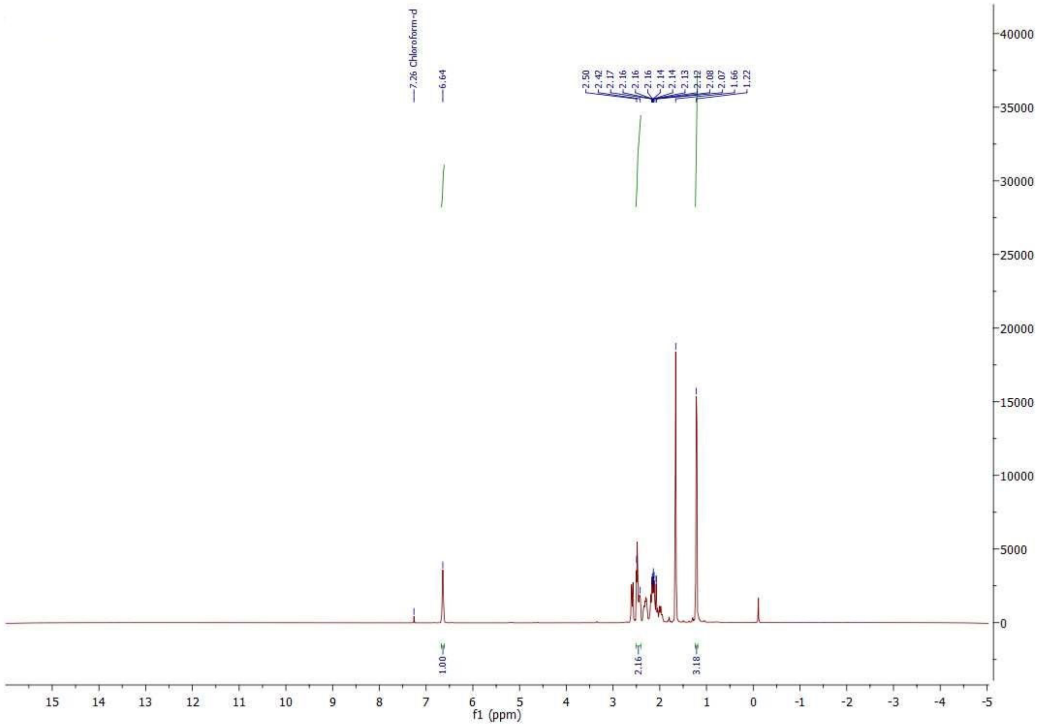

Figure S5. ^{1}H NMR spectrum of **5.**

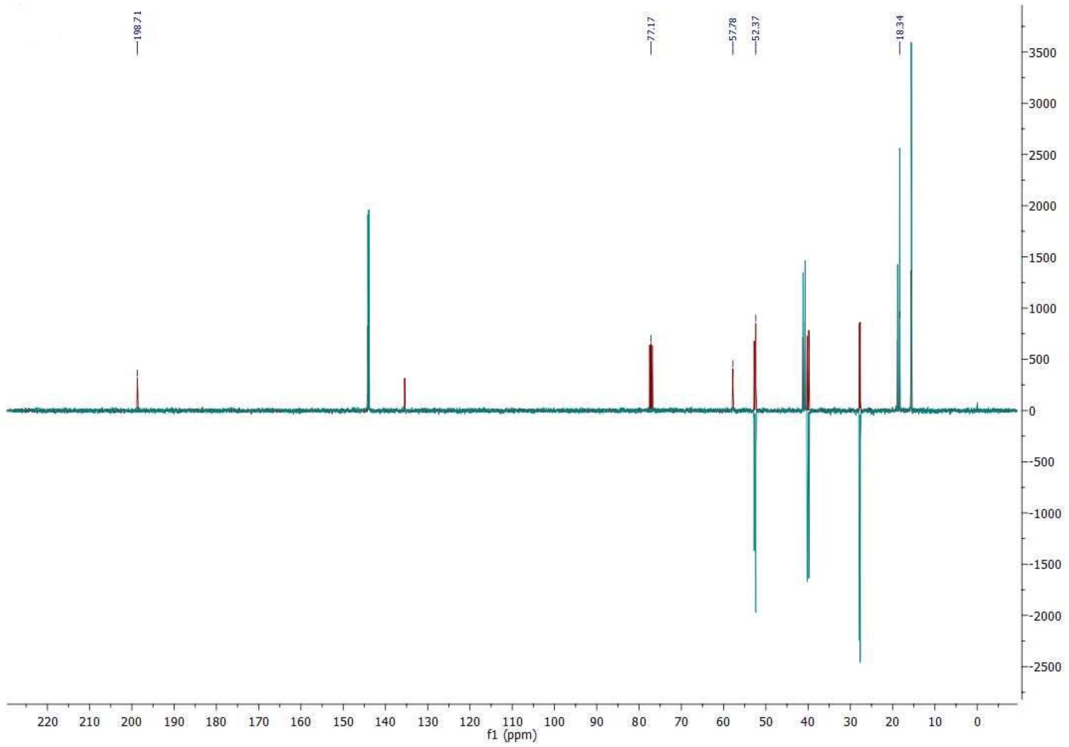

Figure S6. ^{13}C NMR and DEPT-135 spectra of **5.**

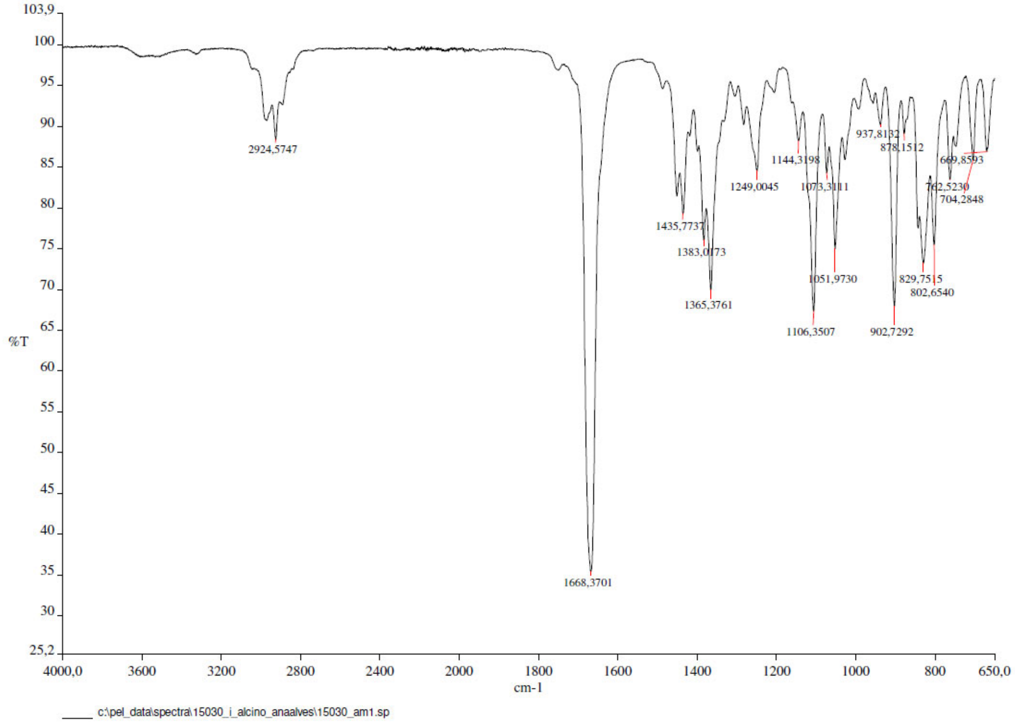

Figure S7. FT-IR spectrum of **5.**

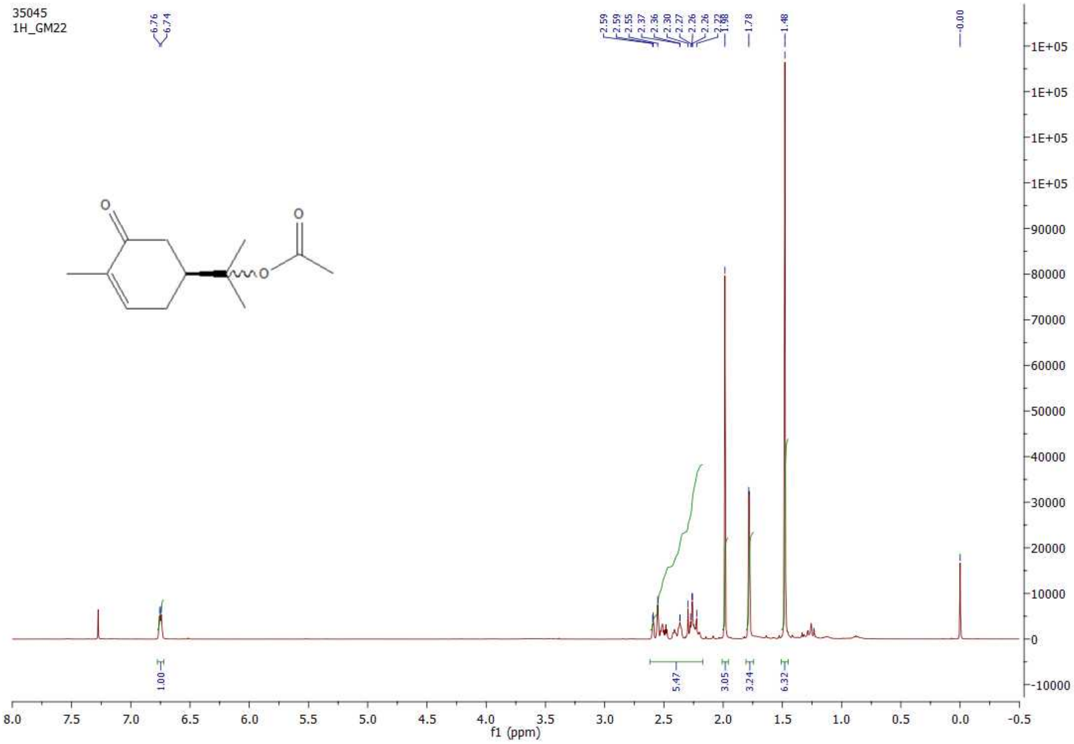

Figure S8. ^{1}H NMR spectrum of **8.**

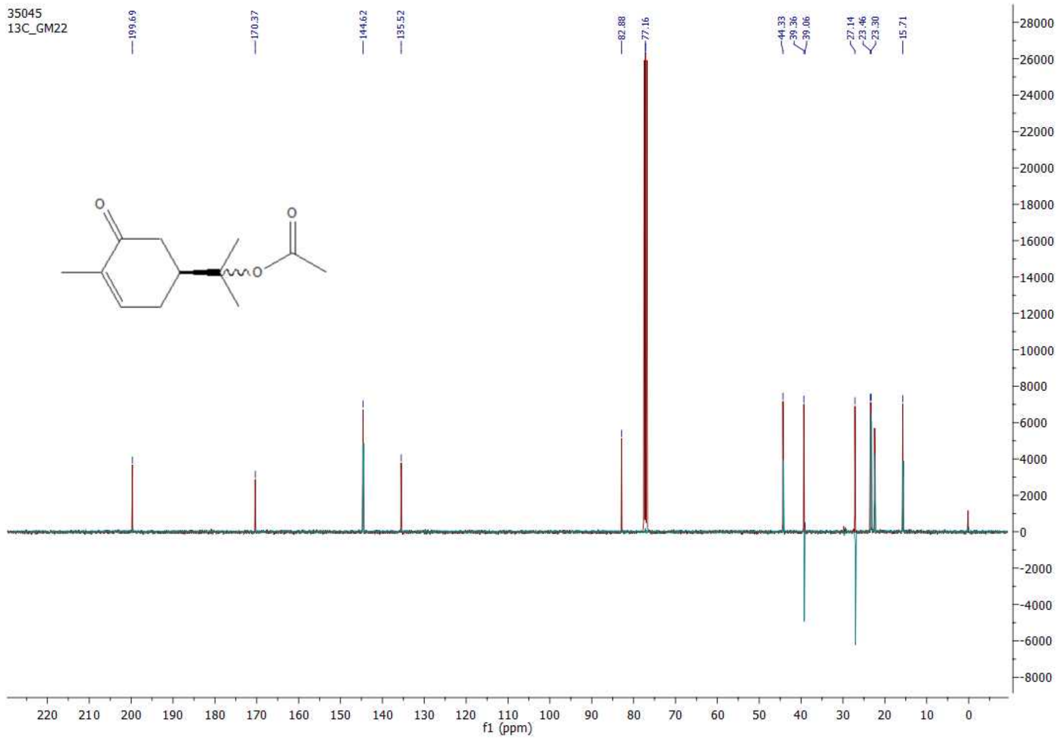

Figure S9. ^{13}C NMR and DEPT-135 spectra of **8.**

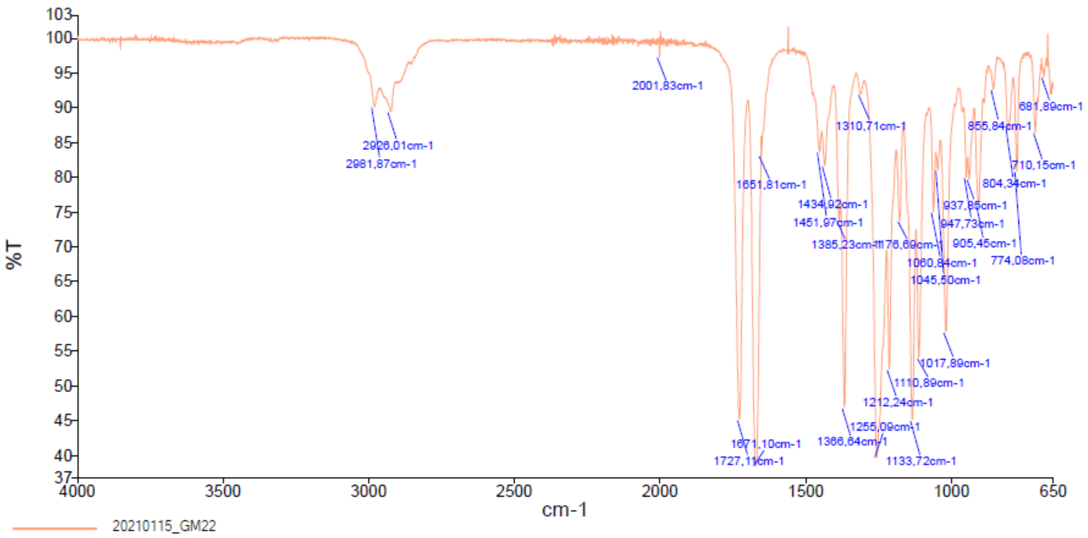

Figure S10. FT-IR spectrum of **8.**

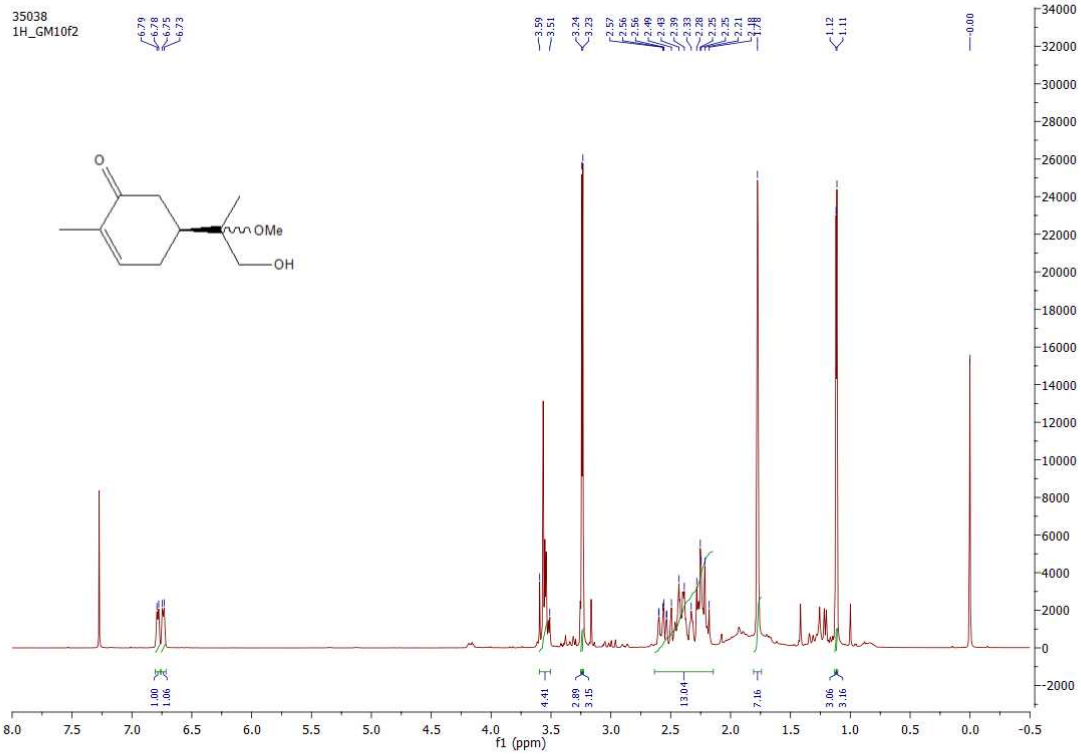

Figure S11. ^{1}H NMR spectrum of **10.**

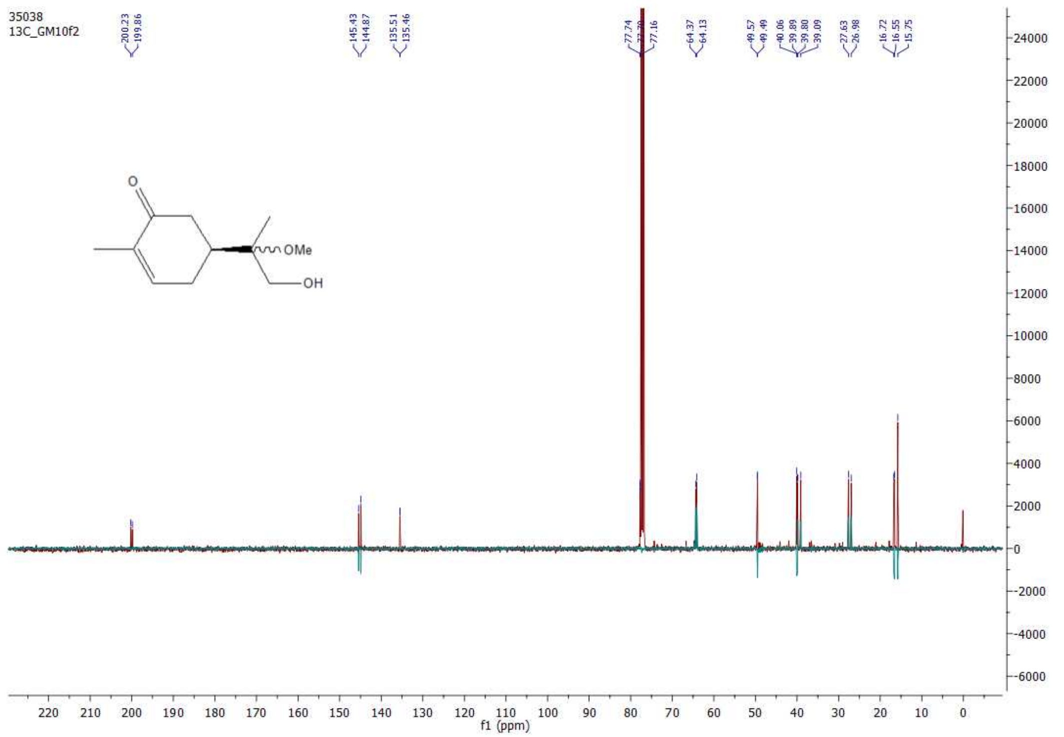

Figure S12. ^{13}C NMR and DEPT-135 spectra of **10.**

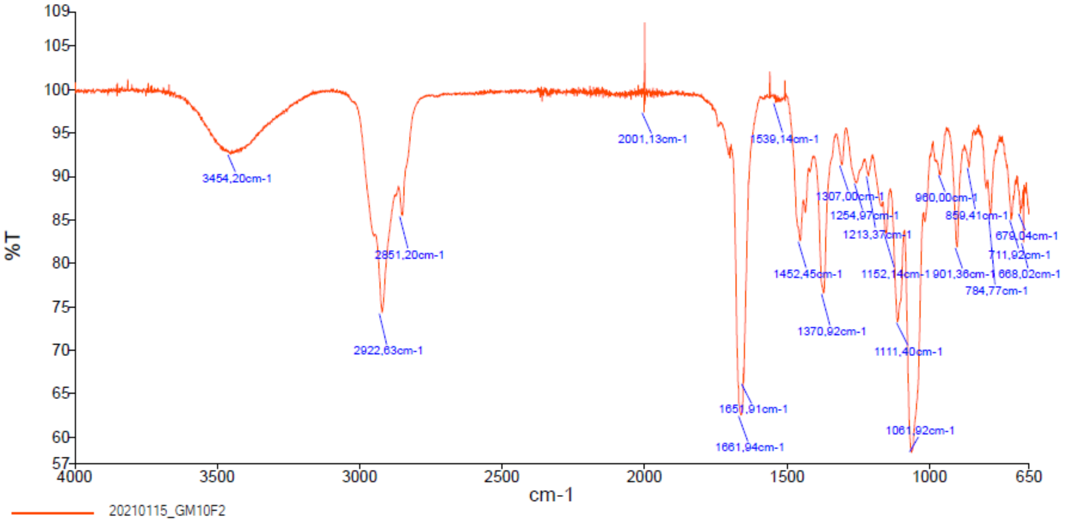

Figure S13. FT-IR spectrum of **10.**

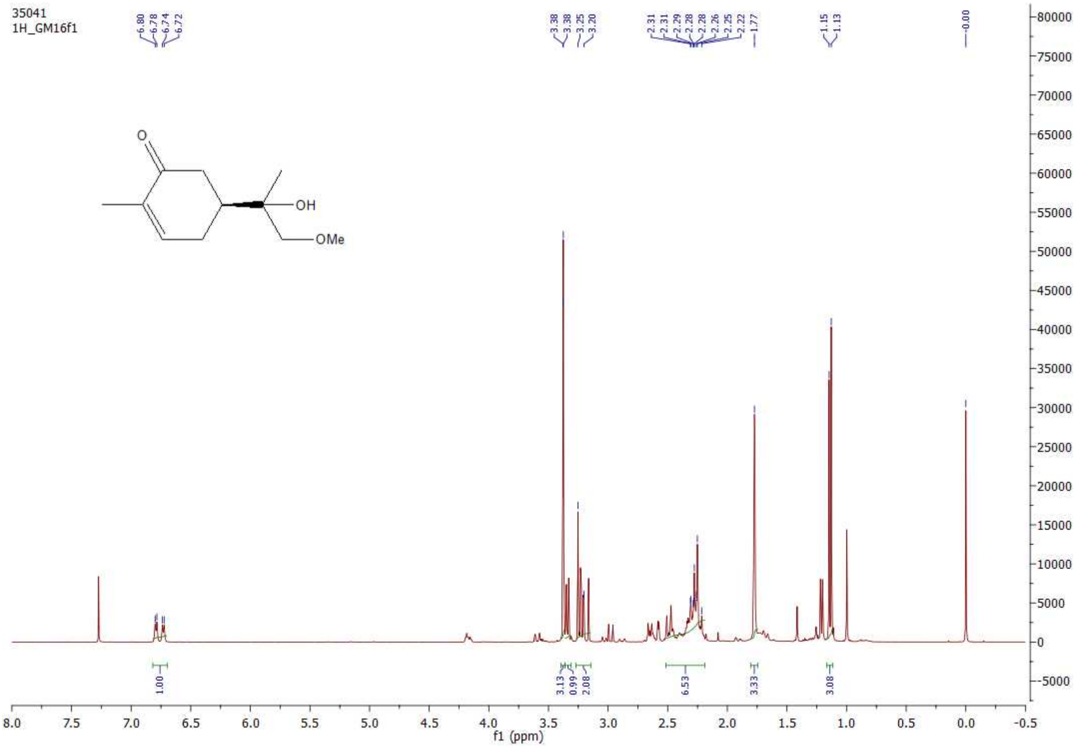

Figure S14. ^{1}H NMR spectrum of **11.**

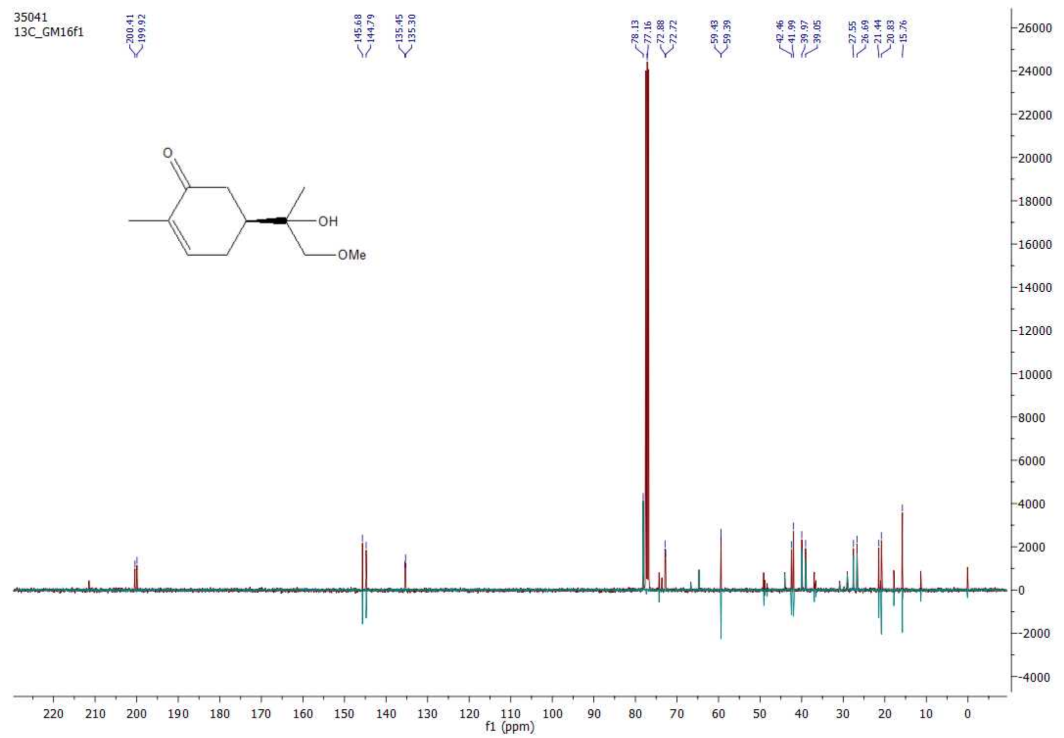

Figure S15. ^{13}C NMR and DEPT-135 spectra of **11.**

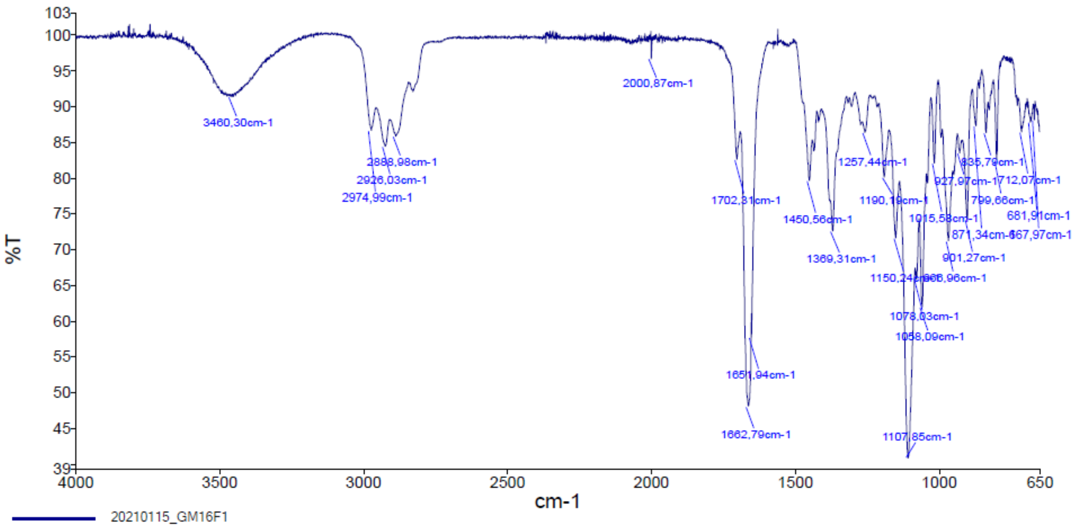

Figure S16. FT-IR spectrum of **11.**

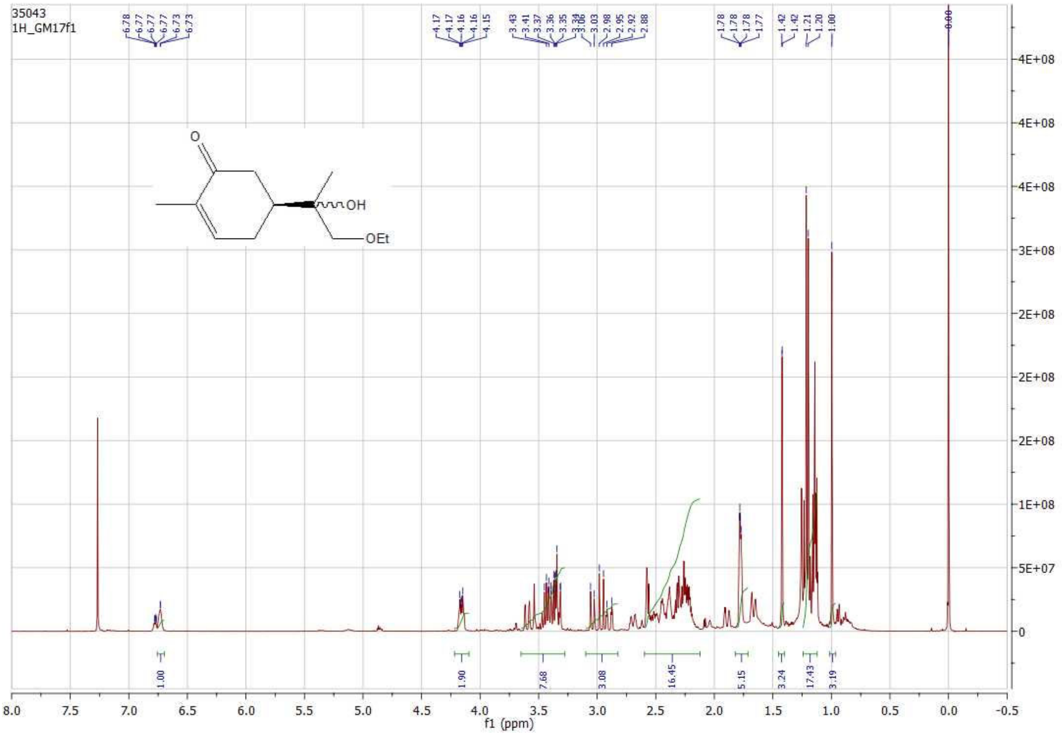

Figure S17. ^{1}H NMR spectrum of **15.**

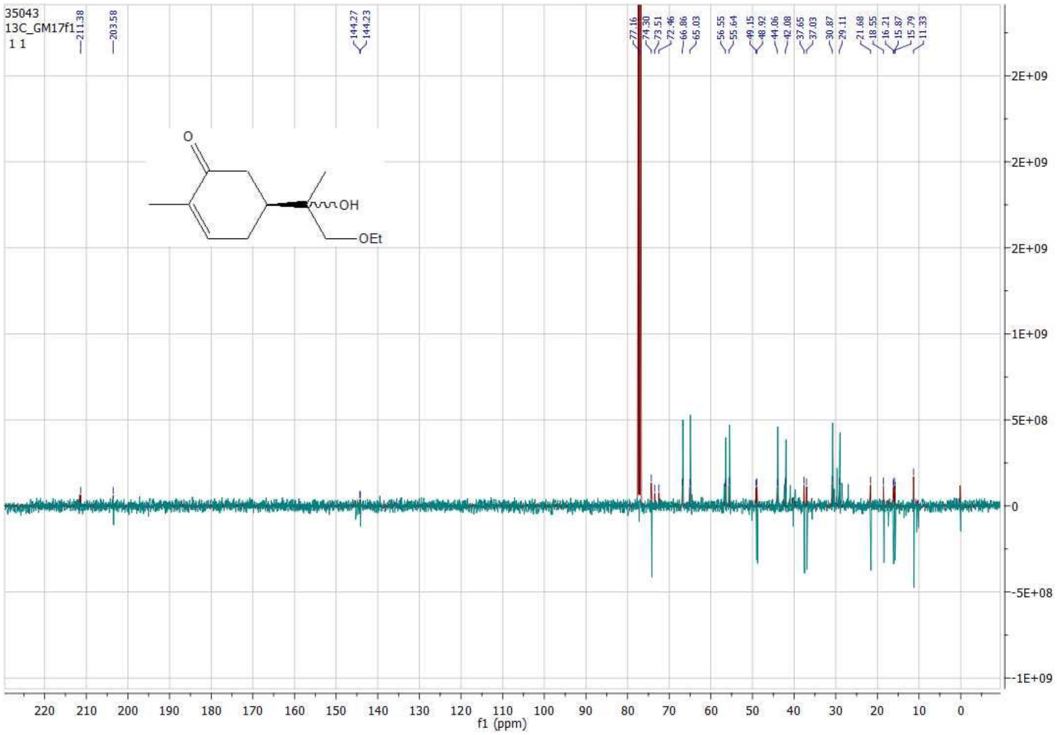

Figure S18. ^{13}C NMR and DEPT spectra of **15.**

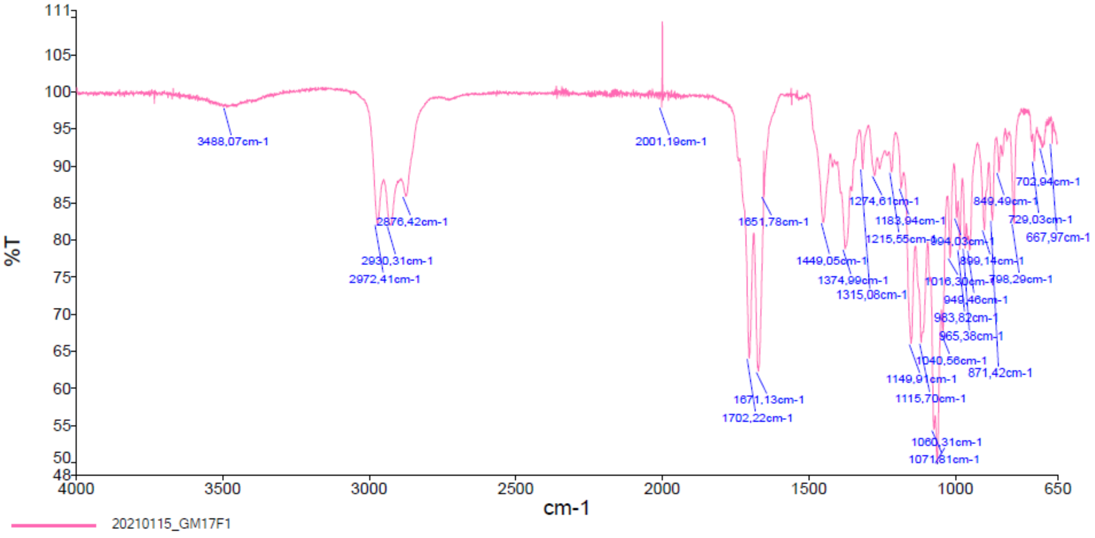

Figure S19. FT-IR spectrum of **15.**

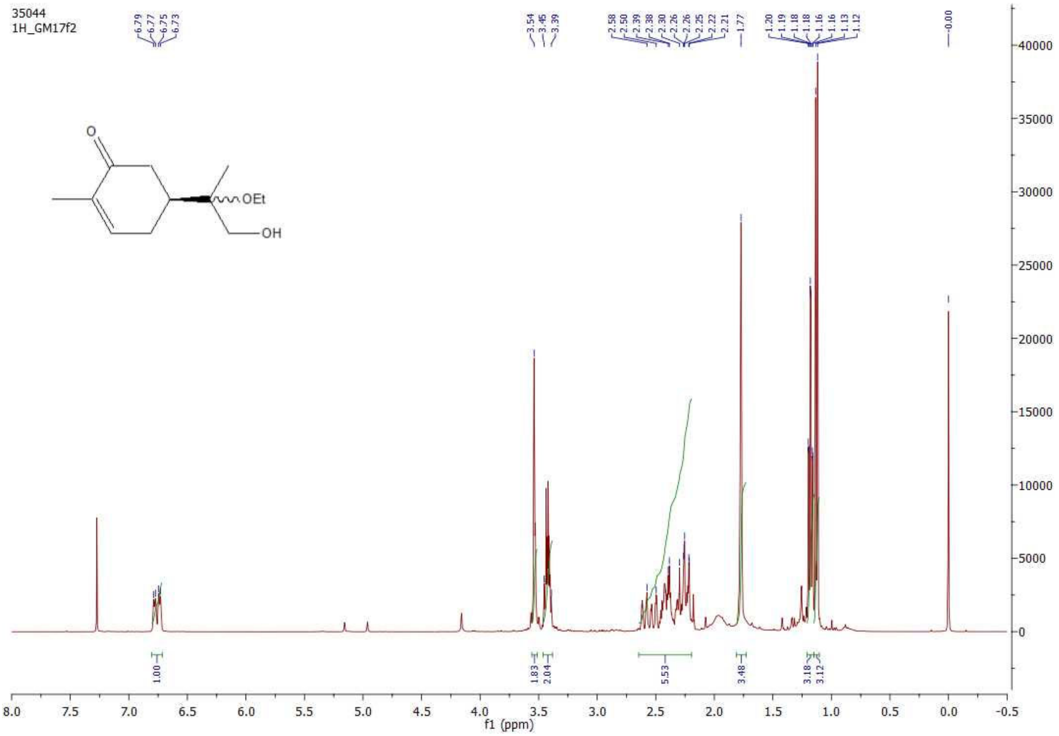

Figure S20. ^{1}H NMR spectrum of **16.**

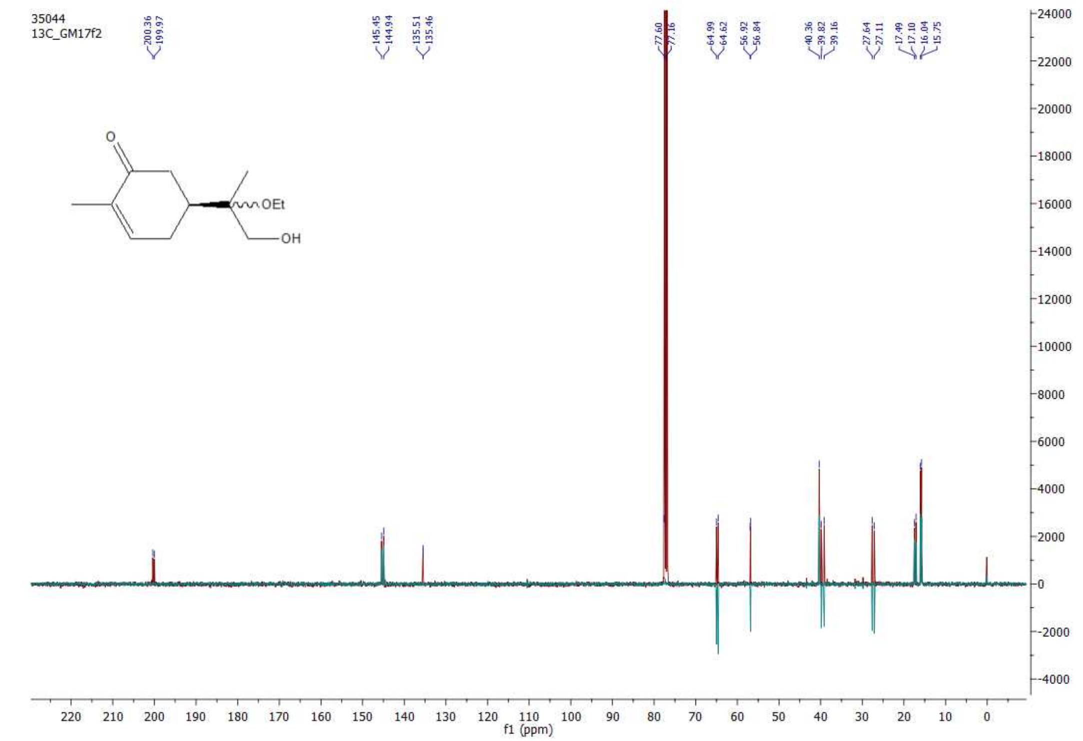

Figure S21. ¹³C NMR and DEPT-135 spectra of **16.**

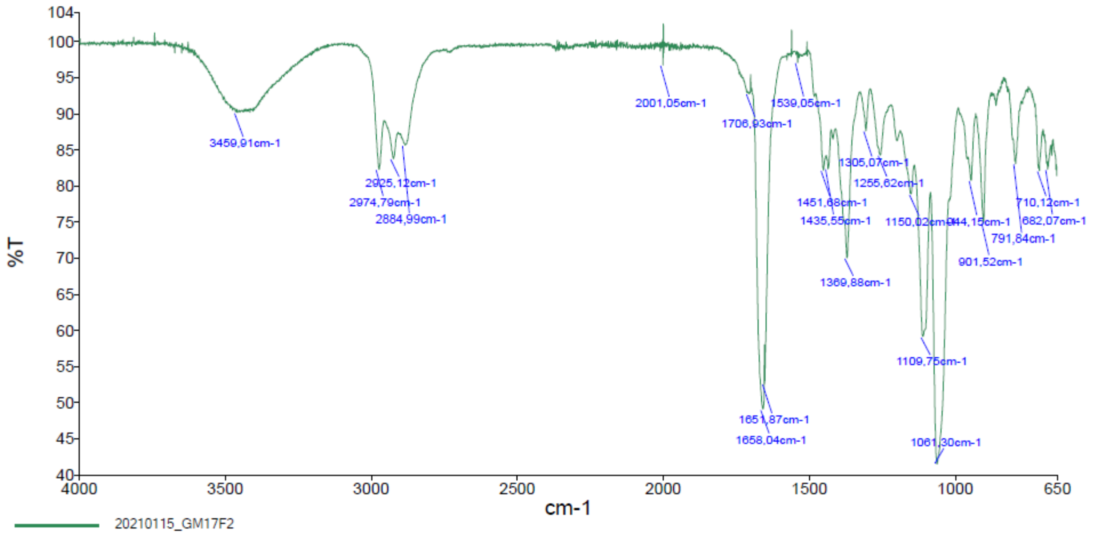

Figure S22. FT-IR spectrum of **16.**

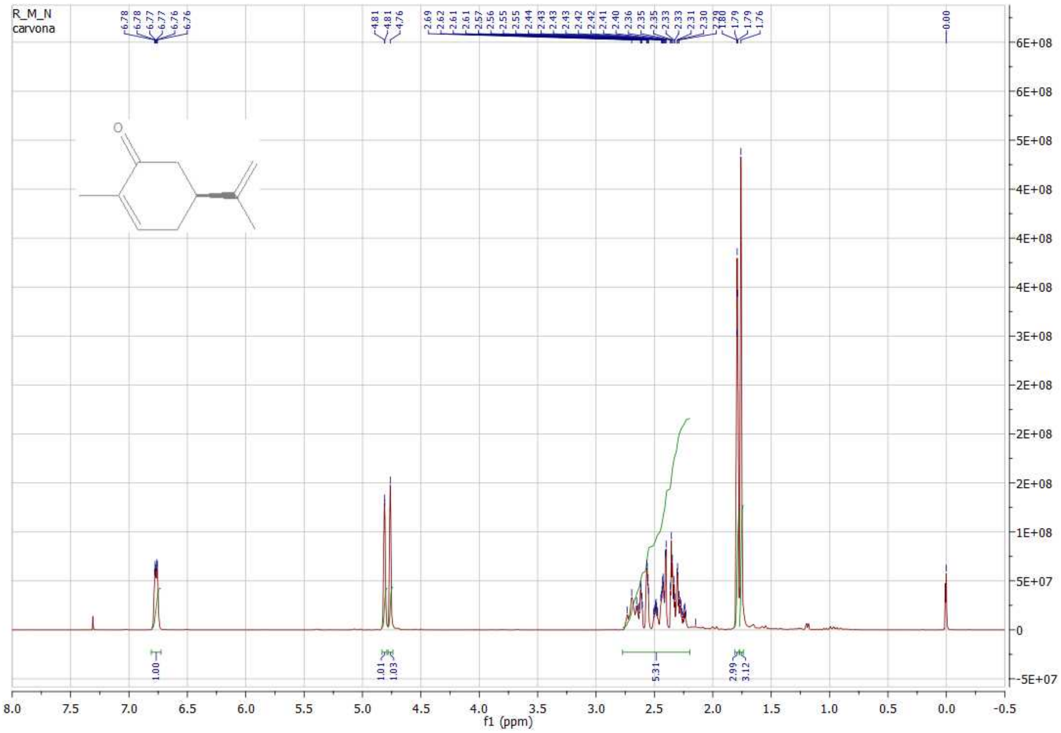

Figure S23. ^{1}H NMR spectrum of Carvone**.**

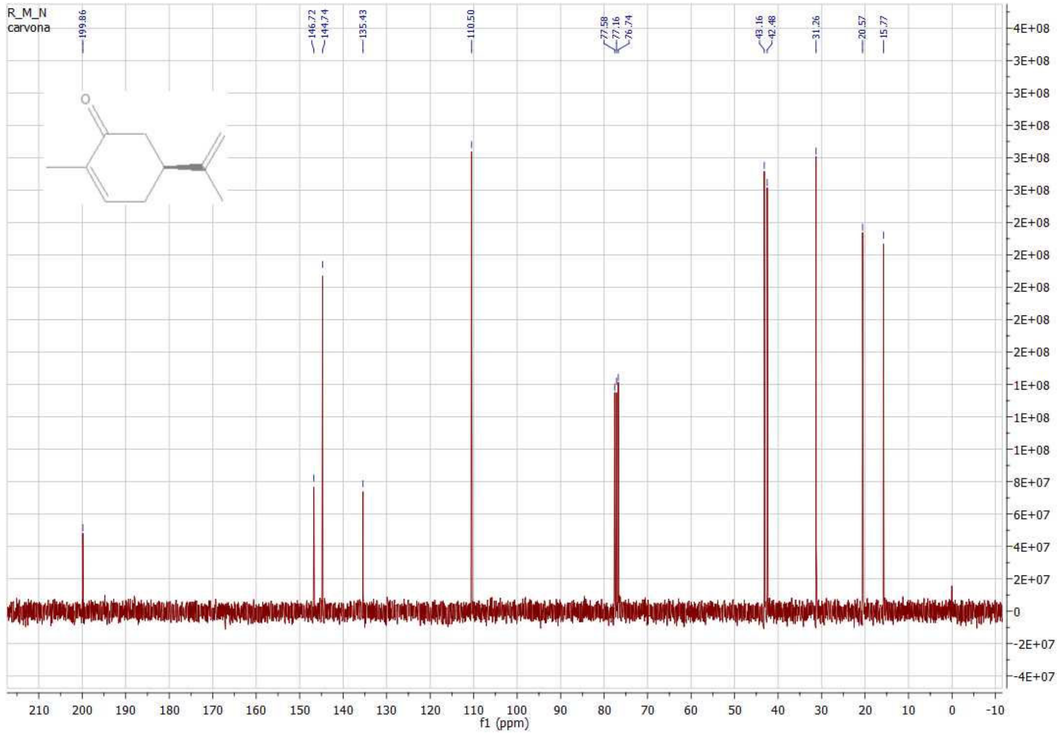

Figure S24. ^{13}C NMR spectrum of Carvone.

Uncropped Blots

Figure S25. Uncropped Blots from Fig. 4A

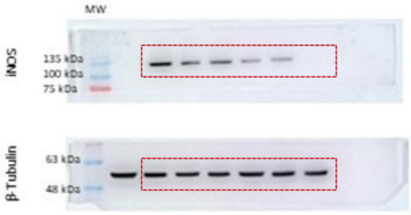

Figure S26. Uncropped Blots from Fig. 4B

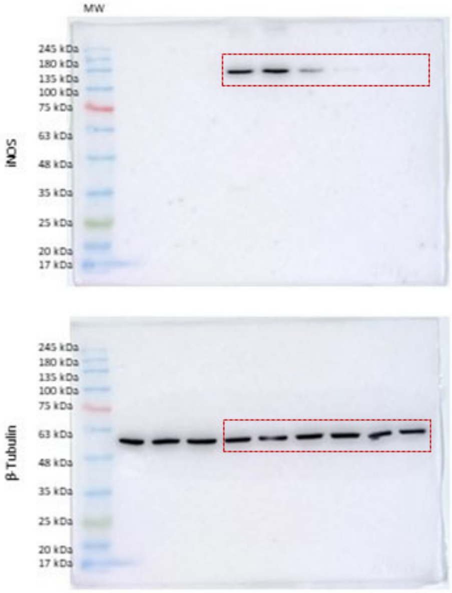

MW: molecular weight marker

Figure S27. Uncropped Blots from Fig. 4C

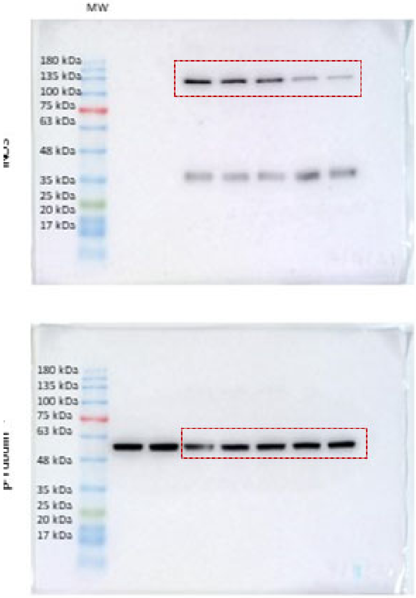

* This membrane is the same shown on Fig. 5C

Figure S28. Uncropped Blots from Fig. 4D

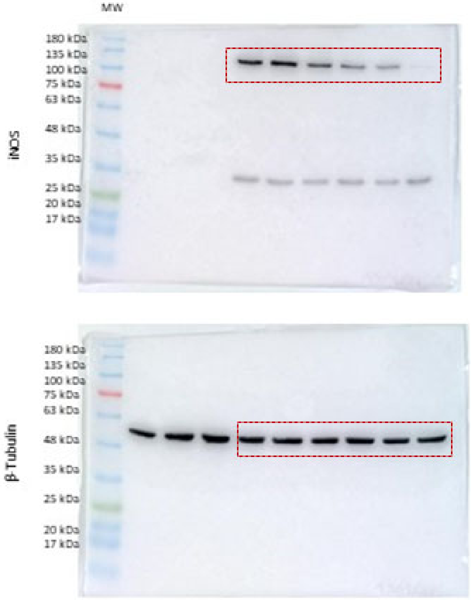

MW: molecular weight marker

The membrane from Fig. 4A was cut at ≈ 68 kDa so that the upper piece was incubated with anti-iNOS antibody and the lower one with the anti-β-tubulin I antibody. The membranes from Figs. 4C and 4D were probed with the anti-iNOS antibody without stripping the anti-pro-IL-1β antibody.

Figure S29. Uncropped Blots from Fig. 5A

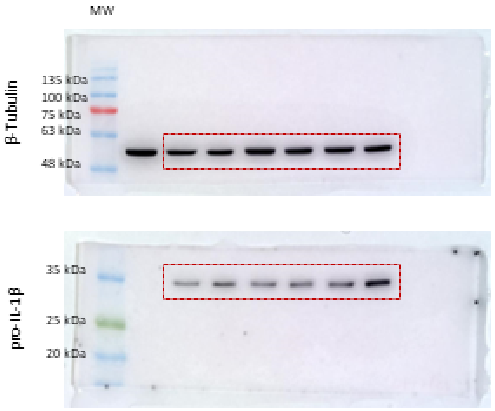

Figure S30. Uncropped Blots from Fig. 5B

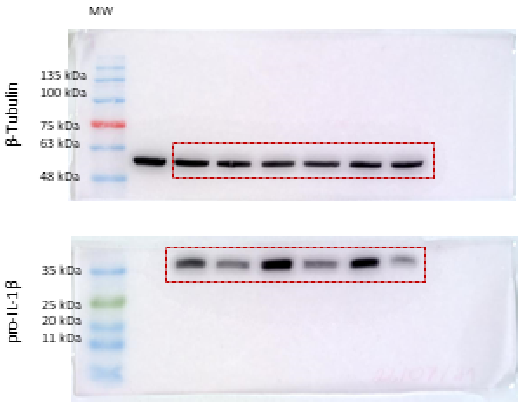

MW: molecular weight marker

The membranes from Fig. 5A and 5B were cut at ≈ 43 kDa so that the upper pieces were incubated with the anti-β-tubulin I antibody and the lower ones with the anti-pro-IL-1β antibody.

Figure S31. Uncropped Blots from Fig. 5C

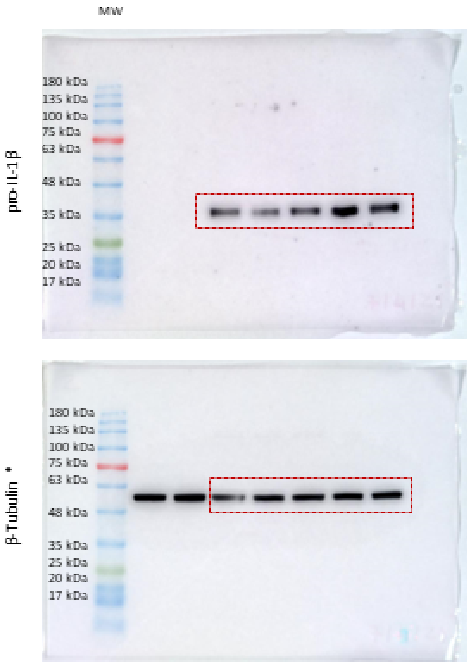

* This membrane is the same shown on Fig. 4C

Figure S32. Uncropped Blots from Fig. 5D

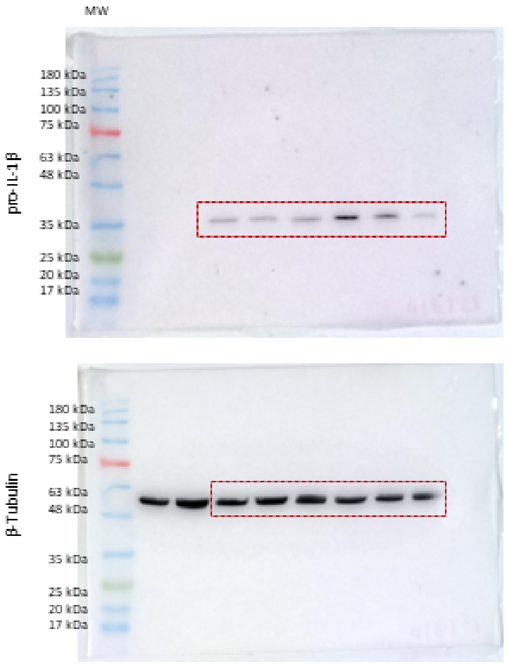

MW: molecular weight marker
